# Supplementary material for: Five-Hour Detection of Intestinal Colonization with Extended-Spectrum-β-Lactamase-Producing Enterobacteriaceae Using the β-Lacta Phenotypic Test: the BLESSED Study
Source: Microbiol Spectr. 2023 Jan 12;11(1):e02959-22. doi: 10.1128/spectrum.02959-22 (PMC9927319; doi:10.1128/spectrum.02959-22)
Supplement: Supplemental file 1 — Supplemental material. Download spectrum.02959-22-s0001.pdf, PDF file, 0.2 MB [file spectrum.02959-22-s0001.pdf]

Five-hour detection of intestinal colonization with extended-spectrum beta-lactamase-producing Enterobacteriaceae using the β-LACTA® phenotypic test: The "BLESSED study"

SUPPLEMENTARY METHODS

Technical optimization procedure

Preliminary phase

A first preliminary clinical phase was conducted directly on fresh rectal swabs without any enrichment or selection phase. Fifty-five consecutive rectal swabs were collected. The β-LACTA® test (BLT) test was performed on bacterial pellets obtained from the centrifugation for 5 minutes at 10,000 *g* of 1 mL of rectal swab medium. Diagnostic performance of this "direct" procedure were then evaluated by comparison with the direct culture of rectal swab medium on the chromID® extended spectrum beta-lactamase (ESBL) plates. The test was positive for 6 out of the 10 ESBL-producing *Enterobacteriaceae* (ESBL-PE) identified in culture, and negative for 26 out of the 45 cultures negative for ESBL-PE. Thus, sensitivity was 60% 95% Confidence Interval (CI) (26%–88%), specificity 58% (42%-72%), positive and negative predictive values 24% (15%-37%) and 86% (75%-94%), respectively. All the 6 ESBL-PE accurately detected by the BLT had an inoculum > 10^5 CFU/mL, while the 4 EBSL-PE not detected by the BLT had lower inoculum < 10^4 CFU/ml.

The poor performance of the BLT in those experimental conditions, attributed to low sensitivity in detecting the inoculum diluted in the swab transport medium on one hand; and by the low specificity due to the false positive result related to cellular and bacterial debris, and, more importantly, to other bacteria that can turn the BLT positive, such as some bacteria of the

anaerobic flora, led us to develop a rapid subculture technique aiming at increasing the ESBL-PE inoculum and at selecting ESBL-PE among all the other components of the digestive flora.

Then, we developed a selective subculture technique to increase both the sensitivity and specificity of the procedure.

Selective enrichment phase

The rapid selective subculture was first validated on rectal samples spiked with calibrated concentrations of ESBL-PE strains. Mock calibrated rectal swab medium suspensions were prepared by mixing 1 mL of eSwab™ liquid medium with 100 mg of a pool of stools documented by selective culture and PCR to be exempt from ESBL and carbapenemase-producing Gram Negative Bacilli (GNB). The mock suspensions were then spiked with two concentrations of ESBL-PE strains collected from different French hospitals (final concentrations in rectal swab medium of 10^3 and 10^4 CFU/mL; 12 different genes [7 CTX-M, 3 TEM and 2 SHV] for 9 different variants). Five hundred microliters of spiked mock rectal swab suspension was added to 4.5 mL of betalactamase-selective enrichment broth (Brain Heart Infusion broth containing an antibiotic mix protected under the patent number IDDN.FR.001.200006.000.S.P.2021.000.31230), and incubated for 2, 3 or 4 h at 37°C under constant agitation. Then, 2 mL of the selective enrichment broth were centrifuged for 5 minutes at 10,000 *g*. The supernatant was discarded, the bacterial pellet was mixed with 1 drop of each BLT-specific reagent, and this final suspension was incubated at 37°C. Results were read after 15, 30, 45 and 60 min by two independent observers. The result was interpreted positive in the case of a colorimetric shift from yellow to orange or red color.

This technical optimization phase allowed us to refine the experimental conditions until the final protocol named "β-LACTA test on Enriched Subcultures of rectal Swabs for ESBL-PE Detection (BLESSED) protocol, combining:

- the best (i.e., providing the highest sensitivity) reading time for the BLT: results are presented in the figure below. The 60-minute delay was then retained in the BLESSED protocol

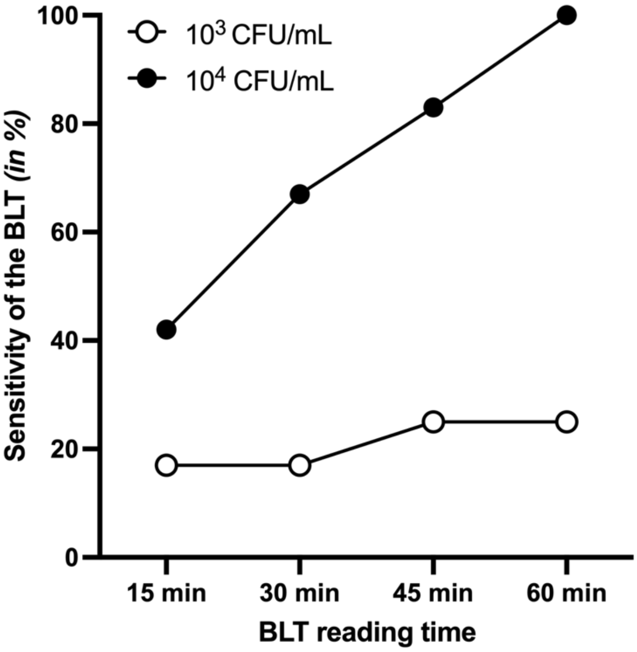

- the best (i.e., providing the highest sensitivity) incubation time in the enrichment selective broth: results are presented in the figure below. The 4-hour incubation time was then retained in the BLESSED protocol as it provided 100% sensitivity for the 10^4 CFU/mL inoculum. Of note, experiments investigating a 5-hour incubation time were not pursued, as maximal sensitivity has already been obtained after 4 hours for the 10^4 CFU/mL inoculum, and as no significant gain in sensitivity was observed for the 10^3 CFU/mL inoculum after the first experiments. Longer incubation time were not evaluated as our goal was to develop a rapid identification protocol for ESBLs that could be easily performed by any laboratory technician in a single work day.

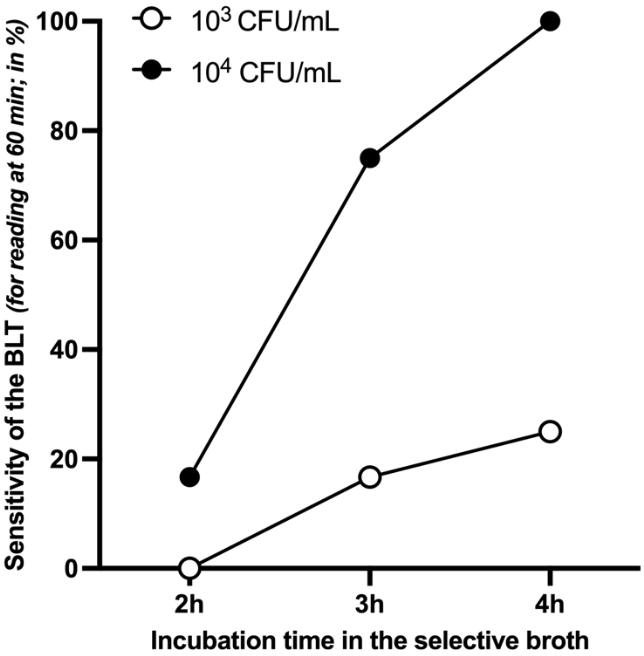

Clinical risk score for ESBL-PE colonization

To assess the relevance of an isolation strategy based on clinical risk factors for ESBL-PE colonization, we compiled nine risk factors from previous studies (1–3) into a clinical risk score : antibiotic therapy received in the last 3 months, previous hospitalization >48h in the last 12 month, intestinal colonization or infection with at least an ESBL-PE strain in the last 6 months, travel in an ESBL endemic zone during the last 12 months, immunosuppression, current hospitalization of more than 5 days prior to admission to intensive care unit, life in institution or nursing home, chronic renal replacement, recurrent urinary tract infection.

References

1. Djibré M, Fedun S, Guen PL, Vimont S, Hafiani M, Fulgencio JP, et al. Universal versus targeted additional contact precautions for multidrug-resistant organism carriage for patients admitted to an intensive care unit. Am J Infect Control. 1 juill 2017;45(7):728‑34.

2. Detsis M, Karanika S, Mylonakis E. ICU Acquisition Rate, Risk Factors, and Clinical Significance of Digestive Tract Colonization With Extended-Spectrum Beta-Lactamase-Producing Enterobacteriaceae: A Systematic Review and Meta-Analysis. Crit Care Med. avr 2017;45(4):705‑14.

3. Mohd Sazlly Lim S, Wong PL, Sulaiman H, Atiya N, Hisham Shunmugam R, Liew SM. Clinical prediction models for ESBL-Enterobacteriaceae colonization or infection: a systematic review. J Hosp Infect. mai 2019;102(1):8‑16.
